# Requirements for guidelines systems: implementation challenges and lessons from existing software-engineering efforts

**DOI:** 10.1186/1472-6947-12-16

**Published:** 2012-03-09

**Authors:** Hemant Shah, Raymond D Allard, Robert Enberg, Ganesh Krishnan, Patricia Williams, Prakash M Nadkarni

**Affiliations:** 1 Henry Ford Health System, Detroit, USA; 2 Center for Medical Informatics, Yale University Medical School, New Haven, USA

## Abstract

**Background:**

A large body of work in the clinical guidelines field has identified requirements for guideline systems, but there are formidable challenges in translating such requirements into production-quality systems that can be used in routine patient care. Detailed analysis of requirements from an implementation perspective can be useful in helping define sub-requirements to the point where they are implementable. Further, additional requirements emerge as a result of such analysis. During such an analysis, study of examples of existing, software-engineering efforts in non-biomedical fields can provide useful signposts to the implementer of a clinical guideline system.

**Methods:**

In addition to requirements described by guideline-system authors, comparative reviews of such systems, and publications discussing information needs for guideline systems and clinical decision support systems in general, we have incorporated additional requirements related to production-system robustness and functionality from publications in the business workflow domain, in addition to drawing on our own experience in the development of the Proteus guideline system (http://proteme.org).

**Results:**

The sub-requirements are discussed by conveniently grouping them into the categories used by the review of Isern and Moreno 2008. We cite previous work under each category and then provide sub-requirements under each category, and provide example of similar work in software-engineering efforts that have addressed a similar problem in a non-biomedical context.

**Conclusions:**

When analyzing requirements from the implementation viewpoint, knowledge of successes and failures in related software-engineering efforts can guide implementers in the choice of effective design and development strategies.

## Background

Numerous papers have proposed or described models and clinical guideline systems (CGS) to support computer-executable guidelines. Comprehensive comparative reviews of different CGSs, notably the reviews of Peleg et al. [1] and de Clerq et al. [2] and the more current review of Isern and Moreno [3] have assisted in defining high-level requirements of guideline systems. In addition, Sweidan et al. [4] deal with evaluation measures associated with clinical software that could be applied to CGSs. However, as the Isern and Moreno review points out, no model appears to have been deployed as production-level software that assists patient management on a daily basis: though one commercial product, Arezzo [5] is based on the PROforma model [6], no peer-reviewed publications describing its use in routine healthcare have appeared to date.

Apart from content-related issues such as guideline quality and comprehensiveness, and their acceptance by clinicians, software-engineering challenges include integration with existing electronic medical record systems (EMRs), and non-clinical hospital software (e.g., scheduling, billing, resource utilization and inventory systems).

Translating the undoubted theoretical advantages of computerized guidelines into better patient care will eventually require switching from models and prototypes to robust operational implementations. To be successful, we believe that such efforts can benefit from careful study of existing software technologies and engineering efforts relevant to CGSs. Benefits include the following:

• System requirements become defined at a much more detailed and granular level, leading in turn to more comprehensive technical specifications. (As defined by Weigers [7], requirements define the "what", while specifications define the "how".) For example, while practically all systems claim to support guideline repositories, production-level sub-requirements include whether and how such repositories support secure access to authorized users, how permissions (read/write vs. read-only, user groups, roles, etc.) are managed, how its content can be searched, and whether it supports version control/audit trail of changes. In existing CGS-related systems, HeCaSe2 [8] has a security model, and the Vaidurya search engine [9] supports search (in part by using standard biomedical vocabularies), but we are not aware of a repository that supports all of the above sub-requirements.)

• Additional requirements may emerge that are of no concern in research prototypes. Such chores rarely constitute "publishable research", but their implementation is essential for production-capable systems. For example

- Security infrastructure related to guideline content and execution must be closely integrated with the EMR within which it is embedded (or with which it communicates, if a separate system): the former cannot be inconsistent with the latter with respect to individual permissions.

- Audit trails must record actions taken by clinicians with respect to individual patients and guidelines at various stages of guideline execution. Clinicians may entirely comply with, ignore, or modestly vary from a guideline: in case of adverse patient outcome, the audit trail may determine legal culpability.

- The system must be highly scalable in terms of ability to deal with large numbers of patients in different stages of varied clinical conditions concurrently.

• Study of successes and failures can provide insight as to which approaches may work (both in the short and long-term), and which may be inadvisable.

• Awareness of general-purpose technologies/tools may allow their repurposing for clinical-guideline infrastructure rather than attempting to create tools *de novo*. Such awareness also helps to determine what tasks or sub-tasks lie within the purview of a guideline framework, and which do not. We refer to such technologies throughout the paper where relevant.

In this paper, we will avoid detailed recapitulation of themes that have been discussed extensively in the guideline literature (e.g., procedural vs. declarative knowledge). If a particular requirement is broadly accepted in the literature, we will simply take it as a given, and cite relevant previous work. We will then focus on sub-requirements that, we believe, have either not been considered in the literature at all (i.e., are novel), or which may have been proposed but whose implications may have not been adequately explored: here, either supporting or (occasional) contrary evidence from existing projects will be presented.

## Methods

The requirements we list in this paper include the following sources:

• A PubMed search of the published requirements listed by authors and developers of different guideline models and systems, as well as guideline-related papers that specify information needs. We used the search term "practice guidelines as topic" [mesh] AND informatics AND "2000"[Publication Date]: "3000" (i.e., to date), which return 350 results (48 reviews), whose titles and abstracts were then used to filter to the topic of interest. Because computer-science conference proceedings are under-represented in PubMed, the search was augmented using Inspec/Engineering Village (which focuses on Engineering and Computer Science) using the term "Clinical guideline" and limiting to publications after 2000.

• Publications in the business workflow domain, which has requirements similar to clinical guidelines, and is mature in terms of operational implementations and third-party add-ons. Inspec was searched for "workflow" in the title and "requirements" in the title/subject/abstract, limited to publications after 2000. 1209 publications were then filtered manually for articles relevant to this paper's theme.

• Our own motivations and wish-list for the Proteus system [10] our experience in its development for over a decade, and its implementation at Henry Ford Health System, within the Semantic Data Capture Initiative project [11].

### Summary of previous comparative reviews

The reviews cited earlier have compared various feature sets of a variety of guideline systems (the systems reviewed varied with the paper). Feature-sets are closely related to requirements: a well-implemented feature in one system that is relevant to the task being addressed becomes a desideratum for future systems. Peleg et al. [1] and Mulyar et al. [12] focused on various dimensions of expressivity; de Clercq et al. [2] dealt with knowledge representation and support for guideline acquisition, verification and runtime execution.

The most recent comparative review, Isern and Moreno [3], addressed a larger variety of themes listed below, which we shall use as the skeleton for the succeeding text. (We have changed their original order, because certain topics are closely related.)

1. Language and knowledge-representation issues (at a summary level).

2. Support for complex coordination.

3. Runtime execution engine

4. Support for distributed execution.

5. Availability of graphical guideline authoring/editing.

6. Support for connectivity with an EMR and non-EMR administrative software (termed "clinical management systems" by the authors)

7. Use of standard terminology or representation language

8. Support for security.

9. Support for guideline repository storage.

### Requirements

We will discuss each theme in the above order (with the exception of security, where we do not have anything special to contribute above what we have briefly explored in the Introduction). The framework for discussion is presented in Table 1.

**Table 1:** Requirements for Guidelines Systems

**Knowledge representation**

Modularity

Support of design patterns

Code base reuse

Technology neutrality

Inferencing-approach neutrality

**Parameterization**

Adapting guidelines to local practices or patient circumstances

Representing non-evidence-based states within guidelines

**Extensibility mechanisms**

**Complex coordination: relation with business workflow systems**

Extensibility

Integration capability

Scalability

Error recovery

Data persistence

Human participation

Auditability

Productive development environment

**Execution**

Execution modes

Support for multiple guideline versions

**Editability**

Collaborative authoring

Requirements traceability

**Integration with existing systems: Use of standards**

**Knowledge maintenance**

Knowledge Life Cycle Management (KLCM)

Guideline repositories

### Knowledge representation

For guideline implementation, support for both declarative and procedural approaches is necessary. Guideline frameworks such as PROforma [6], GLIF [13] and Asbru [14] use a declarative approach (which may or may not use XML syntax) for higher-level specification, while employing an *expression language *(e.g., GELLO [15] for GLIF) for low-level procedural tasks. The specification may be executed by a runtime interpreter or (in the case of PROforma) compiled to code in a general-purpose language (e.g., Prolog) that is then executed by an off-the-shelf interpreter.

One area that we believe has remained under-emphasized in frameworks is the ability to support real-life complexity. Complexity and the solutions that can address it have several related dimensions some of which we address in this section.

#### Modularity

This refers to capabilities within a language that support the development of a large body of code as separate units, possibly by developers working semi-independently, with features such as *namespaces *(which allow different developers to define variables and functions with possibly identical names without having to worry about name-collisions, and *information hiding *[16], where a caller of a routine does not need to know any more than what is passed in and what is returned-allowing the internals of the latter to be modified if needed without any impact on the calling code. Developers using modern programming language take such capabilities for granted, but it is worth remembering that the current official Arden Syntax standard still lacks basic subroutine capability, a feature that FORTRAN has possessed since 1956. This may explain the reluctance of most EMR vendors to support it: it offers few advantages over their proprietary technology. Modularity is supported by CGSs to varying degrees in some frameworks. For example, GLIF and GLARE [17] are intended to support modularity through a distributed architecture while HeCaSe2 supports a distributed architecture of quasi-independent, intercommunicating "agents" or processes.

#### Support of design patterns

Design Patterns [18] are higher-level abstractions that embody algorithmic solutions. An example (implemented in Proteus) is an "at least N" compound-Boolean filter, which yields a result of True if at least N of its input parameters are true: such filters are used in the Jones Criteria for rheumatic fever and for diagnosis of Ventilator Associated Pneumonia (VAP). Peleg and Tu [19] have described the application of design patterns for transformation of clinical guidelines from narrative to executable form.

#### Code base reuse

Reuse of code goes beyond the ability to reuse code written in the language of the CGS itself. One must be able to reuse pre-existing compiled, operational code libraries, even those created elsewhere, without having to rewrite the logic in the guideline/expression language. (With proprietary, compiled code, rewriting may not even be feasible.) For example, vendors provide code that accesses drug databases for drug/drug or drug/laboratory interactions; a variety of dosing algorithms determine drug dosage by adjusting for physiological and pathological states (e.g., age, impaired renal or liver function).

#### Technology neutrality

This implies ability to implement modules using the tool/language most appropriate to the task- this could include not just traditional programming languages, but statistical or mathematical packages, and use them as black-box modules within the larger framework of a guideline.

#### Inferencing-approach neutrality

While certain CGSs, e.g., SpEM [20], rely on production rules [21], alternative approaches exist that may be more appropriate on occasion. These include neural networks [22], decision trees [23], statistical approaches including Bayesian reasoning [24]), and temporal reasoning and representation of temporal uncertainty [25]. Further, these may be employed in the form of commercial black-box libraries or compiled open-source packages.

Many of the above issues are discussed more briefly by Peleg et al. [1]. Issues *2*-4 above come under the rubric of *extensibility*. Today's mainstream programming environments provide extensibility in straightforward ways: in the case of Java, for example, through the Java Native Interface (JNI) [26]. On platforms such as Windows, many libraries are bundled with COM (Common Object Model) or .NET wrappers whose contents (which include skeletal documentation and function parameters) can be browsed within coding environments.

### Parameterization

Two challenges, though seemingly unrelated, could be resolved by allowing a guideline to take parameters, in a fashion similar to subroutines in program code.

#### Adapting guidelines to local practices or patient circumstances

If the modality only allows representing guidelines with all of their details pre-specified, the likelihood of their being used at other locations diminishes. Therefore, some CGSs, e.g., Asbru, support the notion of *Goal *or *Intention *for activities [27]. An example would an activity with the goal "administration of ACE inhibitor to lower blood pressure by at least 10 mmHg", while leaving the medication regime details (ACE agent, dose) to the clinician.

#### Representing non-evidence-based states within guidelines

Sutton et al., in an evaluation of PROforma [9] also advocate the ability to "conceal inessential details" by allowing variation within a guideline (e.g., an observation period of N weeks, where N, a parameter, is a number that can be institution/user-specified. (PROforma currently lacks such a capability.) Such flexibility is required because of absence of conclusive evidence in literature, so that certain decisions are best left to clinician-users.

Many general-purpose programs (notably tax-computation software) support parameterized customization through mechanisms such as templates or wizards, and future CGSs should support parameterization of parts of guidelines similarly. Batet et al. [28] describe a CGS for home care services that supports both modularization (using a distributed architecture) and parameterization with respect to individual intervention plans.

### Extensibility mechanisms

The CGS guideline-definition framework should allow guideline definition at *different abstraction levels*. For example, the subject-matter experts can define the guideline at a relatively high, generic level: lower-level details (e.g., interfacing with external systems) can be left as placeholders to be locally customized. For example, in some programming languages, production software libraries achieve such designs through mechanisms such as *callbacks*, where hooks are provided within the library at critical points for developers to provide their own functions. (A very early example was the *sort*() function in the C language, which took as a parameter a developer-supplied sorting function.)

### Complex coordination: relation with business workflow systems

The high-level-design operations provided in guideline languages closely resemble those in "workflow languages", which are intended to address complex coordination. A workflow is a machine-interpretable model of a "progression of steps (tasks, events, interactions) that comprise a work process, involve one or more persons, and create or add value to the organization's activities." [29] Mulyar et al. [12] have in fact evaluated guideline languages in depth with respect to their support of a variety of workflow coordination patterns, which were originally characterized by van der Aalst et al. [30].

A workflow is an extension of a flowchart, with nodes representing activities (tasks) or decision points and the edges represent flow of control (e.g., branching, looping) or sequencing of activities. The extension comes from the ability to specify that certain operations may execute in parallel, or that certain activities (or a certain number of activities) must complete before a decision point can be evaluated, in addition to operations, e.g., suspend, delay or terminate an operation, etc. (Individual packages may also support specific operations such as sending e-mail, writing data, etc.) The graphical nature of workflows makes them a natural fit for a visual-programming metaphor, though a machine-friendly counterpart (typically based on an XML dialect) also exists.

Business workflow frameworks are fairly mature and widely used products. Standards such as the XML-based Business Process Execution Language for Web Services (WS-BPEL) [31] and Business Process Markup Notation (BPMN) [32] are supported by vendors such as Oracle, HP and IBM, and tend to be programmer-oriented. Microsoft Office 2010 SharePoint Server [33], enterprise integration software such as Microsoft BizTalk and Enterprise Resource Planning (ERP) systems such as SAP [34] include proprietary workflow languages with workflows intended to be authored primarily by non-programmer domain experts (e.g., business analysts). Microsoft provides native workflow support within the Windows operating system through Windows Workflow Foundation [35]: WWF workflows can be authored in Visual Studio.

The study of business-workflow technology provides important lessons for CGS implementers because it deals efficiently with many issues that CGS implementers, have yet to address systematically. Below, we summarize these issues: the parallels to patient-care workflow are obvious.

### Extensibility

Extensibility allows developers to add to the capabilities of the base software through custom code written in one's programming language of choice. Workflows are generally executed in interpreted mode by a runtime workflow-execution engine. However, they provide an Application Programming Interface (API) that allows developers to implement custom tasks (with well-defined inputs and outputs) using traditional languages. The compiled code is added to the authoring environment's toolbox, to be reused in any workflow. There are very few limits on what custom tasks may do: Vadaparty [36] describes a custom-task set for WWF created at Merrill Lynch that invokes multiprocessor parallel hardware over a network to execute complex finance-related tasks in a manner transparent to the non-programmer workflow author.

#### Integration capability

Integration refers to the ability of the system to inter-operate with external software packages. These systems allow communication with external systems by a variety of methods, including ODBC and Web services (which may run on multiple hardware platforms). CGSs need to inter-operate with Electronic Health Record (EHR) and Computerized Provider Order Entry systems in order to access (and optionally modify) patient-related data.

#### Scalability

This refers to the ability of the system to execute a large number of instances (workflows, guidelines) concurrently without noticeable performance degradation. Business workflow engines routinely execute thousands of diverse workflows that are critical to business operations, and can invoke parallel hardware.

#### Error recovery

The systems are capable of recovery from hardware- or network-related error conditions. Design of Web-service-based workflow can allow for *resource unavailability*, and *synchronization/communications failures*.

#### Data persistence

Persistence of Data is the ability to create and retrieve data that outlives the software process that created it (i.e., when the hardware is shut down for maintenance). Workflow software uses DBMS technology to persist workflow state: CGSs may also use their own data stores (e.g., to store electronic guidelines), but should ideally store patient-related data in an EHR where possible.

#### Human participation

The workflow systems allow devising algorithms that rely on human input in a decision-making capacity. Certain tasks involve human actions (e.g., loan approval sign-off by one or more geographically distributed persons). Certain steps may allow for human override in case of unforeseen circumstances, sometimes through a consultative process. This capability is particularly important in medicine: numerous papers, e.g., Hurwitz [37], have highlighted the reluctance among clinicians to use clinical guidelines because of the fear that they will not be in charge.

#### Auditability

The ability to maintain an electronic audit trail includes logging of context and human actions takes place at all decision points, especially human overrides, much as alert overrides in Computerized Physician Order Entry (CPOE) systems are logged.

#### Productive development environment

Mature authoring, validation and testing tools are integral to sound workflow systems. The more complex a workflow, the more likely are errors of consistency (e.g., circular or conflicting logic) to be introduced in its design. Many commercial and open-source tools, notably for WS-BPEL, support inconsistency diagnosis through *static analysis *and logical-error taxonomies [12], validation (e.g., ActiveVOS [38] and testing (e.g., Oracle BPEL Test Framework [39]).

While Greenes [40] notes the complexity of workflow management, Fox et al. [41] emphasize that workflow-management, decision-making and care-planning (all of which are involved in complex guidelines) are aspects of clinical work that clearly benefit from computer support. Fox et al. in fact point out that BPMN is a good fit for defining plans formally.

Finally, there is nothing intrinsically unique to the "business" domain about many business-workflow engines: WS-BPEL and BPMN are in fact general-purpose, and their use for clinical applications deserves serious exploration. One way to do this is for CGSs to "compile" guideline definitions into a general-purpose workflow language, with medicine-specific functionality being implemented as calls to custom tasks. This would allow the numerous vendor/third-party tools to be leveraged without needing to re-invent the numerous wheels necessary to make CGSs production-grade. It might also simplify integration with administrative systems, many of which utilize BPEL (or are BPEL-ready): The current version of BPMN (version 2) is also accumulating open-source support (e.g., Activiti [42] and jBPM [43],

### Execution

#### Execution modes

CGSs need to operate in one of two modes:

1. Simulated (testing) mode. Most CGSs support some form of testing, in the form of tracing through the guideline's different paths. Certain clinical consultation systems (e.g., QMR [37]) implement this capability comprehensively, to serve as a teaching aid.

2. Online Mode. Here the guideline operates with actual patients, either *interactively *in a dialog with the user, in *batch *on a set (or population) of patients, or unobtrusively as an event-driven *agent *activates on a particular pattern of inputs. (Alerting mechanisms in CPOEs operate in the last fashion: as stated earlier, HeCaSe2 has an agent-based architecture.)

During interactive mode, the ability to provide *explanation of actions *is important, as suggested by Tolchinsky et al. [44] and Fox et al. [45], with the text of explanations customized to the user role. More advanced capabilities would include the ability of the inferencing mechanism to learn (through machine-learning algorithms) from the inferences and decisions that the clinicians make in different situations.

#### Support for multiple guideline versions

Unlike most decision-support systems, a patient going through a guideline workflow may stay within that workflow for long periods. For example, a workflow related to management of infertility could last more than a year, because pregnancy takes time to establish. The clinical aspects of the workflow may no longer apply (e.g., because of the availability of a new and better treatment, recommendations may have changed), and so a new clinical sub-workflow may supersede the old one.

However, workflows also have administrative aspects (inventory adjustment, service itemization, reimbursement from the insurer/payer), whose management can be more complex: for different patients, multiple versions of the same administrative sub-workflow may run simultaneously. For example, even if a reimbursement agreement has changed since the patient has begun treatment, the contract with the payer may require the old agreement to stay in force.

Therefore, depending on the nature of the guideline/workflow, the execution engine may need to enforce one of two strategies:

• Replace the old version with the current version for all instances/patients (This is technically challenging because many patients are in the middle of a flow, and there must be rules that specify what is to be done if, for example, a patient is in a guideline branch that is now obsolete.)

• Allow multiple versions of the same broad workflow to coexist and run at the same time, the choice of version depending on the patient.

The knowledge representation employed by advanced business-workflow engines supports hierarchical versioning, with an individual version represented as a "child" of a base version, and differences between the base and child modeled as atomic change units analogous to the "deltas" used in software version control [46], along with the metadata at the base level that specifies which of the above strategies is to be followed if changes occur. Every active instance is associated with its current version, and its current position/branch within the version, and the path taken to get to this position. The runtime execution engine will implement the desired strategy for a given instance if changes occur to the guideline. While most engines use proprietary approaches, there are attempts, e.g., Juric et al. [47], to apply such techniques to standard representation languages such as WS-BPEL.

### Editability

Isern and Moreno point out that most CGSs incorporate visual (graphical) editors for guideline authoring. However, these editors are not available freely, as open-source or otherwise, making their usability and robustness difficult to evaluate. User interfaces that are optimal for novices may be less productive for advanced users, who might prefer rapid typing to manipulating graphical icons [19], so usable interfaces could allow switching between text and graphical mode, with *round-trip engineering *[20]: modification of code leads to changes in the graphic, and vice versa.

In text mode, high-productivity Integrated Development Environments (IDEs) such as Eclipse™ and Microsoft Visual Studio™ offer aids such as auto-completion, auto-indent, outlining with collapsing/expansion of code segments, color-based syntax highlighting macros to improve typing productivity, and browsing of class/code libraries. Given that these tools have a large user base, and that they are highly customizable by developers (e.g., by incorporation of plug-ins), CGS developers would be well advised to leverage such tools as the basis for authoring environments.

Adequate documentation to teach and promote best practices for guideline authoring and maintenance are also desirable.

#### Collaborative authoring

The Isern and Moreno review suggests the possibility of supporting Web-based editing and collaborative authoring by diverse subject-matter experts: CGSs currently lack these features. While software that supports Web conferencing (e.g., Citrix GotoMeeting [48], Mikogo [49] is widely used, collaborative authoring that supports an orderly change process with conflict resolution and change trail maintenance is challenging to implement well; early versions of Google Wave [50], for example, suffered from poor user acceptance.

Another practical problem is that many IDE productivity features for both textual and graphical editing are difficult to achieve in the presence of a network communication delay. In the more than seven years of their existence, both the Eclipse and Visual Studio IDEs have remained desktop-based. (Google Docs, which supports collaborative editing of the same document, has 30-second refresh latency before another person's edits can be seen.) One approach is to augment Web-based editing with instant messaging and voice chat, e.g., Ajax.org's Cloud9 project [51], and to permit only one writer per file (e.g., Borland CodeWright), thereby minimizing the risk of conflicting edits.

One aspect of collaborative authoring is achievement of consensus. Deshpande et al. [52] have described Web support of Delphi rating, a well-known consensus-development methodology, which was deployed prior to the Conference for Guidelines Standardization.

#### Requirements traceability

Sutton et al., in their evaluation of PROforma [53], emphasized the future need for *structure preservation*: the models used in knowledge acquisition should be identical or highly similar to those used for design and implementation. This is similar to *requirements traceability *[9], a software-engineering process to ensure that a software product's implemented features remain synchronized with previously defined requirements.

#### Integration with existing systems: use of standards

The need for CGSs to communicate bi-directionally (i.e., read/write) with EMR and administrative systems well established. Several lessons have been learned regarding integration for Decision Support Systems (DSS) deployment in clinical and business contexts (e.g., Enterprise Resource Planning (ERP).

Incomplete integration is worse than no integration at all: by increasing the user's cognitive burden as to which steps are electronic and which remain manual, perceived workload is increased rather than reduced.

DSS is successful to the extent that it is non-obtrusive and reduces caregiver workload, so that the default action is the desired one, and takes little or no human effort to perform. Miller et al. [54] point out in the context of consultation systems that the failure to embed them as integral components of normal clinician workflow impaired their acceptability. Effective embedding relies on adequate integration.

Thus, a system smart enough to recognize what tasks need execution should be smart enough to perform those tasks if possible, relying on users only for confirmation/override; e.g., a useful reminder system goes beyond telling the physician to schedule a vaccination or order a glycated hemoglobin, and volunteers to schedule or order it, unless the physician says no, through communication with the scheduling system.

A practical problem is that, because integration standards are immature (HL7 vMR or virtual Medical Record [55]), not widely accepted by all vendors (HL7 v3) or non-existent (links to administrative systems), CGS implementers must work with proprietary vendor APIs, and extensive mapping of guideline elements to elements in the local electronic systems must be performed manually, as described by Peleg et al. [56] and Isern et al. [57]: the latter used the ontology editor Protégé [58] for mapping. Both efforts utilize standard biomedical vocabularies (UMLS in the latter case.) The SEBASTIAN decision-support framework of Kawamoto and Lobach [59] uses Web services to partially insulate the guideline framework from the specifics of individual APIs [60]. Ongenae et al. [61] describe a system that utilizes a rule-based engine (built with Drools [62]) combined with standard medical controlled vocabularies and representation languages.

### Knowledge maintenance

#### Knowledge life cycle management (KLCM)

In the context of decision support, a knowledge artifact is a discrete entity created by author(s) that contributes toward behavior changes in the software based on its knowledge. The artifacts may evolve from a hazy concept, expressed in plain text, to an actionable form, and finally into something that can be encoded into the software application to realize its purpose, moving progressively from one state to another. The knowledge artifacts and their associated metadata are the main constituents of knowledge repositories. KLCM is the ability to maintain knowledge artifacts current and accurate from inception to retirement.

In the clinical guideline area, several papers have dealt with different aspects of KLCM, such as creation and evaluation (Cecamore et al. [63]), content refinement (Haynes and Wilczynski [64]), analysis of barriers to implementation (Goud et al. [65]), sharing (Paterno et al. [66]), and repository design (the Morningside initiative [67]).

Agile methods are a group of software engineering approaches which emphasize short iterations (2-6 weeks) of development with simultaneous ongoing automated and user-testing so that the software's evolution remains closely aligned to the user expectations [68]. The agile methodologies potentially bear on KLCM, because a guideline can be verified and tested even as it is being fleshed out, allowing rapid iterations of refinement.

#### Guideline repositories

We have addressed some of the sub-requirements of guideline repositories in the Introduction: security and management of user privileges, version control and audit trail of changes, and searchable content (possibly assisted by controlled vocabularies). High-end version-control systems possess all of the above capabilities built in (including text-word search, but not vocabulary-assisted search). However, many commercial systems (e.g., Visual Studio Team Foundation Server) and open-source systems (Apache Subversion) have programmable APIs that can be utilized to provide extensibility: several third-party authoring environments (e.g., Adobe RoboHelp [69], a tool for creation of online documentation) "plug in" into these systems.

It is possible that repository indexing of the searchable content may be augmented by user-specified terms. The system should also allow leveraging the user community as a resource for feedback about quality and errors: most version systems are closely coupled with feature/defect tracking subsystems (e.g., Bugzilla [70] for open-source projects).

## Conclusion

The contribution of this paper is two-fold:

• It discusses in detail the sub-requirements that become critical during implementation of production clinical guideline systems, in accordance with the framework outlined in Table 1. These have not been previously discussed in the literature to the requisite depth needed by production-CGS implementers.

• It cites practical issues from a closely related, but far more mature domain, that of business-workflow engines, whose implementers have encountered, and often solved, many of the problems that CGS implementers have yet to encounter because the latter have not attempted to create industrial-strength systems that are used in daily patient care. Production-capable CGSs must demonstrate the same robustness as the CPOE and EHR software with which they are required to inter-operate.

We believe that a study of successes in the broader software-development world can help point the way. Noting the occasional failures is also important: to quote George Santayana's dictum, those who refuse to learn the lessons of history are condemned to repeat it. Therefore, rather than simply stating that a sub-requirement is desirable, we have drawn attention to specific examples, which we hope, will be useful to future CGS developers.

## Competing interests

The authors declare that they have no competing interests.

## Authors' contributions

This work was primarily done by HS who is also the Co-Principal Investigator of the Semantic Data Capture Initiative (SDCI) Project within which this work was partly accomplished. RDA and RE were the PIs of the SDCI project during different periods. GK was a software developer in the SDCI project. PW was the analyst and project manager of the SDCI project. PMN was the external advisor for the SDCI project and contributed significantly to the authorship of this paper. All authors read and approved the final manuscript.

## Pre-publication history

The pre-publication history for this paper can be accessed here:

http://www.biomedcentral.com/1472-6947/12/16/prepub

## References

[B1] Peleg M Tu S Bury J Ciccarese P Fox J Greenes RA Hall R Johnson PD Jones N Kumar A Comparing computer-interpretable guideline models: a case-study approach J Am Med Inform Assoc 2003 10 52 68 10.1197/jamia.M1135 12509357 PMC150359

[B2] de Clercq PA Blom JA Korsten HH Hasman A Approaches for creating computer-interpretable guidelines that facilitate decision support Artif Intell Med 2004 31 1 27 10.1016/j.artmed.2004.02.003 15182844

[B3] Isern D Moreno A Computer-based execution of clinical guidelines: a review Int J Med Inform 2008 77 787 808 10.1016/j.ijmedinf.2008.05.010 18639485

[B4] Sweidan M Williamson M Reeve JF Harvey K O'Neill JA Schattner P Snowdon T Evaluation of features to support safety and quality in general practice clinical software, BMC Medical Informatics and Decision Making 11 (2011), 27 BMC Medical Informatics and Decision Making 2008 2008 8 48 8 48

[B5] Arezzo http://www.infermed.com/index.php/arezzo

[B6] Sutton DR Fox J The syntax and semantics of the PROforma guideline modeling language J Am Med Inform Assoc 2003 10 433 443 10.1197/jamia.M1264 12807812 PMC212780

[B7] Wiegers K Software requirements 2003 Redmond, WA: Microsoft Press

[B8] Isern D Sánchez D Moreno A Burkhard H-D, Lindemann G, Verbrugge R, Varga LZ HeCaSe2: A Multi-agent Ontology-Driven Guideline Enactment Engine Multi-Agent Systems and Applications V 4696 Berlin, Heidelberg: Springer Berlin Heidelberg 322 324

[B9] Moskovitch R Shahar Y Vaidurya: a multiple-ontology, concept-based, context-sensitive clinical-guideline search engine J Biomed Inform 2009 42 11 21 10.1016/j.jbi.2008.07.003 18721900

[B10] Shah H Proteus-A Model for Clinical Protocols Created from Knowledge Components Proceedings of the Fourteenth IEEE Symposium on Computer-Based Medical Systems 2001 Washington, DC, USA: IEEE Computer Society 59 64

[B11] Shah H Krishnan G Williams P Vogler A Allard RD Nadkarni PM Interoperability and Integration Considerations for a Process-Oriented Clinical Decision Support System 2011 IEEE World Congress on Services 2011 Washington DC, USA: IEEE Computer Society, Los Alamitos, California, USA 437 442

[B12] Mulyar N van der Aalst W Peleg M A Pattern-based Analysis of Clinical Computer-interpretable Guideline Modeling Languages Journal of the American Medical Informatics Association 2007 14 781 787 10.1197/jamia.M2389 17712087 PMC2213484

[B13] Boxwala AA Peleg M Tu S Ogunyemi O Zeng QT Wang D Patel VL Greenes RA Shortliffe EH GLIF3: a representation format for sharable computer-interpretable clinical practice guidelines J Biomed Inform 2004 37 147 161 10.1016/j.jbi.2004.04.002 15196480

[B14] Seyfang A Miksch S Marcos M Combining diagnosis and treatment using ASBRU Int J Med Inform 2002 68 49 57 10.1016/S1386-5056(02)00064-3 12467790

[B15] Sordo M Ogunyemi O Boxwala AA Greenes RA GELLO: an object-oriented query and expression language for clinical decision support AMIA Annu Symp Proc 2003 1012 14728515 PMC1480304

[B16] Booch G IEEE Trans Softw Eng Object-oriented development 1986 12 211 221

[B17] Terenziani P Molino G Torchio M A modular approach for representing and executing clinical guidelines Artif Intell Med 2001 23 249 276 10.1016/S0933-3657(01)00087-2 11704440

[B18] Gamma E Helm R Johnson R Vlissides J Design Patterns 1995 Reading, MA: Addison-Wesley

[B19] Peleg M Tu S Design patterns for clinical guidelines Artif Intell Med 2009 47 1 24 10.1016/j.artmed.2009.05.004 19500956

[B20] Dube K Mansour E Wu B Supporting collaboration and information sharing in computer-based clinical guideline management Proceedings of 18th IEEE Symposium on Computer-based Medical Systems 2005 2005 CBMS,: Dublin, Ireland. IEEE Press 232 237

[B21] de Vries PH de Vries Robbé PF: An overview of medical expert systems Methods Inf Med 1985 24 57 64 3889542

[B22] Baxt WG Application of artificial neural networks to clinical medicine Lancet 1995 346 1135 1138 10.1016/S0140-6736(95)91804-3 7475607

[B23] Podgorelec V Kokol P Stiglic B Rozman I Decision trees: an overview and their use in medicine J Med Syst 2002 26 445 463 10.1023/A:1016409317640 12182209

[B24] Ashby D Bayesian statistics in medicine: a 25 year review Stat Med 2006 25 3589 3631 10.1002/sim.2672 16947924

[B25] Adlassnig K-P Combi C Das AK Keravnou ET Pozzi G Temporal representation and reasoning in medicine: research directions and challenges Artif Intell Med 2006 38 101 113 10.1016/j.artmed.2006.10.001 17081736

[B26] Java Native Interface http://en.wikipedia.org/wiki/Java_native_Interface

[B27] Shahar Y Miksch S Johnson P An intention-based language for representing clinical guidelines Proc AMIA Annu Fall Symp 1996 592 596 8947735 PMC2233124

[B28] Batet M Isern D Marin L Martínez S Moreno A Sánchez D Valls A Gibert K Knowledge-driven delivery of home care services Journal of Intelligent Information Systems 2010 38 95 130

[B29] What is workflow? definition and meaning http://www.businessdictionary.com/definition/workflow.html

[B30] van der Aalst W ter Hofstede AHM Kiepuszewski B Barros A Workflow Patterns Distributed and Parallel Databases 2003 14 5 51 10.1023/A:1022883727209

[B31] Static analysis requirements for WS-BPEL http://docs.oasis-open.org/wsbpel/2.0/OS/wsbpel-v2.0-OS.html#_B._Static_Analysis_requirement

[B32] BPMN Information Home http://www.bpmn.org/

[B33] English B The Administrator's Guide to SharePoint Portal Server 2010 2010 Boston, MA: Addison-Wesley

[B34] SAP Business Workflow http://www.sdn.sap.com/irj/sdn/index?rid=/webcontent/uuid/10ff0453-ae33-2a10-7984-9d8df609d8f9

[B35] The Workflow Way: Understanding Windows Workflow Foundation http://msdn.microsoft.com/en-us/library/dd851337.aspx

[B36] Vadaparty K Multithreaded Parallelism in Windows Workflow Foundation MSDN Magazine 2008

[B37] Hurwitz B Legal and political considerations of clinical practice guidelines British Medical Journal 1999 318 661 664 10.1136/bmj.318.7184.661 10066215 PMC1115098

[B38] ActiveVOS: A business process automation platform for IT project teams http://www.activevos.com/

[B39] Oracle BPEL Test Framework http://download.oracle.com/docs/cd/E12483_01/integrate.1013/b28981/testsuite.htm

[B40] Greenes Re Clinical Decision Support: The Road Ahead 2007 New York: Academic Press/Elsevier

[B41] Fox J Glasspool D Patkar V Austin M Black L South M Robertson D Vincent C Delivering clinical decision support services: there is nothing as practical as a good theory J Biomed Inform 2010 43 831 843 10.1016/j.jbi.2010.06.002 20601124

[B42] Activiti BPM Platform http://www.activiti.org

[B43] JBoss BPM Platform (jBPM) http://www.jboss.org/jbpm

[B44] Tolchinsky P Cortés U Grecu D Annicchiarico R, Cortés U, Urdiales C Basel Argumentation-Based Agents to Increase Human Organ Availability for Transplant Agent Technology and e-Health Birkhäuser Basel 65 93

[B45] Fox J Glasspool D Grecu D Modgil S South M Patkar V Argumentation-based inference and decision making-a medical perspective IEEE Intell Syst 2007 22 34 41

[B46] Krinke J Zeller A Linux/Unix Programming Toolset: Version Control, Construction, Testing, and Debugging 2001 New York, NY: John Wiley

[B47] Juric M Sasa A Rozman I WS-BPEL extensions for versioning Information & Software Technology 2009 51 1261 1274 10.1016/j.infsof.2009.03.003

[B48] GoToMeeting http://www.gotomeeting.com

[B49] Mikogo http://www.mikogo.com

[B50] Google Wave http://wave.google.com

[B51] Cloud9: your cloud anywhere, anytime http://c9.io

[B52] Deshpande AM Shiffman RN Nadkarni PM Metadata-driven Delphi rating on the Internet Comput Methods Programs Biomed 2005 77 49 56 10.1016/j.cmpb.2004.05.006 15639709

[B53] Sutton DR Taylor P Earle K Evaluation of PROforma as a language for implementing medical guidelines in a practical context BMC Med Inform Decis Mak 2006 6 20 10.1186/1472-6947-6-20 16597341 PMC1459125

[B54] Miller R Masarie F The demise of the Greek oracle model of diagnostic decision support systems Meth Inform Med 1990 29 1 8 2407929

[B55] Virtual Medical Record (vMR)-HL7Wiki http://wiki.hl7.org/index.php?title=Virtual_Medical_Record_(vMR)

[B56] Peleg M Keren S Denekamp Y Mapping computerized clinical guidelines to electronic medical records: knowledge-data ontological mapper (KDOM) J Biomed Inform 2008 41 180 201 10.1016/j.jbi.2007.05.003 17574928

[B57] Isern D Sanchez D Moreno A Ontology-driven execution of clinical guidelines Comput Methods Programs Biomed 2011 10.1016/j.cmpb.2011.06.006 21752487

[B58] Noy NF Crubezy M Fergerson RW Knublauch H Tu SW Vendetti J Musen MA Protégé-2000: an open-source ontology-development and knowledge-acquisition environment AMIA Annu Symp Proc 2003 953 14728458 PMC1480139

[B59] Kawamoto K Lobach DF Design, implementation, use, and preliminary evaluation of SEBASTIAN, a standards-based Web service for clinical decision support AMIA Annu Symp Proc 2005 380 384 16779066 PMC1560495

[B60] Borbolla D Otero C Lobach DF Kawamoto K Gomez Saldaño AM Staccia G Lopez G Figar S Luna D Bernaldo de Quiros FG Implementation of a clinical decision support system using a service model: results of a feasibility study Stud Health Technol Inform 2010 160 816 820 20841799

[B61] Ongenae F De Backere F Steurbaut K Colpaert K Kerckhove W Decruyenaere J De Turck F Towards computerizing intensive care sedation guidelines: design of a rule-based architecture for automated execution of clinical guidelines BMC Medical Informatics and Decision Making 2008 2010 8 48 10 13 10.1186/1472-6947-10-3 PMC2823596 20082700

[B62] JBoss Enterprise Business Rules Management System http://jboss.com/products/rules

[B63] Cecamore C Savino A Salvatore R Cafarotti A Pelliccia P Mohn A Chiarelli F Clinical practice guidelines: what they are, why we need them and how they should be developed through rigorous evaluation Eur J Pediatr 2011 170 831 836 10.1007/s00431-010-1344-y 21132571

[B64] Haynes RB Wilczynski NL Effects of computerized clinical decision support systems on practitioner performance and patient outcomes: methods of a decision-maker-researcher partnership systematic review Implement Sci 2010 5 12 20181104 10.1186/1748-5908-5-12 PMC2829489

[B65] Goud R van Engen-Verheul M de Keizer NF Bal R Hasman A Hellemans IM Peek N The effect of computerized decision support on barriers to guideline implementation: a qualitative study in outpatient cardiac rehabilitation Int J Med Inform 2010 79 430 437 10.1016/j.ijmedinf.2010.03.001 20378396

[B66] Paterno MD Maviglia SM Ramelson HZ Schaeffer M Rocha BH Hongsermeier T Wright A Middleton B Goldberg HS Creating shareable decision support services: an interdisciplinary challenge AMIA Annu Symp Proc 2010 2010 602 606 21347049 PMC3041453

[B67] Greenes R Bloomrosen M Brown-Connolly NE Curtis C Detmer DE Enberg R Fridsma D Fry E Goldstein MK Haug P The morningside initiative: collaborative development of a knowledge repository to accelerate adoption of clinical decision support Open Med Inform J 2010 4 278 290 21603282 10.2174/1874431101004010278 PMC3097479

[B68] Larman C Agile and iterative development: a manager's guide 2004 Boston: Addison-Wesley

[B69] Adobe RoboHelp http://www.adobe.com/products/robohelp.html

[B70] Bugzilla: a defect-tracking system http://www.bugzilla.org

